# A grammatico-pragmatic analysis of the *because* X construction: Private expression within public expression

**DOI:** 10.12688/f1000research.72971.1

**Published:** 2021-09-24

**Authors:** Masaru Kanetani

**Affiliations:** 1 Faculty of Humanities and Social Sciences, University of Tsukuba, Tsukuba, Japan

**Keywords:** Because X construction, private expression, public expression, construction grammar, three-tier model of language use, metapragmatic strategy

## Abstract

**Background:** This article investigates an innovative use of *because*, called the *because* X construction (e.g., because homework). Quantitative and qualitative research as well as research about the historical development of the construction have been conducted. The present article aims to determine what motivates the use of the construction.

**Methods:** Based on the data collected from the literature and online sources, the grammar of the *because* X construction is described in detail. The construction is then analyzed  within Hirose’s (2015) three-tier model of language use.

**Results:** A two-layered expressive structure is proposed: The X-element serves as a private expression, which is a speaker’s expression of thought with no intention of communication, whereas the whole construction functions publicly. The private nature of the X-element consistently accounts for the syntactic categories of the X-element and the restrictions on them observed in the literature.

**Conclusion:** The proposed two-layered expressive structure reflects a metapragmatic function of the construction. A private, subjective expression embedded in a public expression has the function of connecting the hearer to the speaker, and it accordingly brings about a joint attention effect. With such a function, the proposed structure is effective especially (but not exclusively) in online communication because one can strategically indicate closeness or intimacy to others, particularly in an environment where nonverbal means are difficult to apply.

## 1. Introduction

The word *because* in English, which is typically followed by a finite clause or an *of* phrase, conveys a cause or reason, as exemplified in (1a, b):

(1) a. He’s not coming to class because he’s sick.
b. He’s not coming to class because of his sickness.

An innovative use of *because* has recently emerged that is in use particularly in online communication and colloquial conversation in which a single word or non-clausal phrase directly follows *because*, as exemplified in (2):

(2) I cannot go out with you today because homework/sick.

As single words in various grammatical categories such as nouns, adjectives, and interjections can follow *because*, the construction is called the *because* X construction. This article investigates the characteristics of this construction, focusing particularly on the status of the X-element from the perspective of the three-tier model of language use (e.g., Hirose 2015). Specifically, it is claimed in accordance with Kanetani (2017) that the X-element serves as a private expression, namely, a speaker’s expression of thought with no intention of communication, although the whole construction is used publicly. ^1^ Pragmatic effects in response to the proposed structure are also discussed.

The present article is organized as follows. After outlining the research methodology in section 2, section 3 observes the semantic and syntactic properties of the construction. Section 4 investigates various ways in which the X-elements are construed as private expressions. Section 5 reviews the typological characteristics of English from the perspective of the three-tier model of language use. Sections 6 and 7 explore the motivations for these private expressions.

## 2. Methods

The analysis in the present article follows a traditional linguistic methodology. First, the grammar of the target constitution is described in detail from both semantic and syntactic points of view based on the grammaticality or acceptability of linguistic data, which are collected from the literature and online sources including tweets, blogs, and corpora. As the *because* X construction has only recently emerged and come to be recognized, the availability of descriptive data from research papers is limited and the description of grammar is not sufficient. Therefore, describing the semantic and syntactic characteristics of the construction based on the collected data is essential for analyzing the construction.

After the grammar is described, the construction is analyzed within a certain theoretical framework, and discussions about theoretical implications follow. Specifically, the construction is analyzed by revising and expanding Kanetani’s (2016, 2017, 2019) account of the *because* X construction from a perspective of the three-tier model of language use, which claims that the X-element functions as a private expression while the entire construction functions as a public expression. The three-tier model is a grammatico-pragmatic theory developed by Hirose (2013, 2015, 2016) as a natural extension from the notion of private and public expressions ( Hirose 2000). While the details of the model will be introduced in section 5, it should be noted here that the grammatico-pragmatic characteristics of a language can only be defined relatively to other languages. In fact, the three-tier model ascribes differences between languages to different combination patterns of the three tiers of language use, namely, the *situation construal*, *situation report*, and *interpersonal relationship* tiers. Therefore, to highlight the grammatico-pragmatic traits of the English language, it is compared to Japanese and the markedness of the construction within the system of English grammar is emphasized.

However, my earlier analyses left open the question of what motivates the proposed expressive structure of the construction. Therefore, comparing the *because* X construction with other linguistic phenomena of similar expressive structures (an innovative use of *kudasai* ‘please’ in online communication ( Naya 2017) and soliloquy insertion in conversations ( Hasegawa 2010)), the present article discusses the significance of the proposed structure and a general metapragmatic strategy behind these expressions.

## 3. The *because* X construction

Kanetani (2015, 2016, 2017, 2019) treats the phenomenon from the perspective of construction grammar, where a construction is generally defined as a conventionalized pairing of form and function ( Fillmore *et al*. 1988; Goldberg 1995; Hoffman and Trousdale 2013, among many others). After briefly reviewing the functional properties of the construction in section 3.1, I identify the formal properties in section 3.2 and then describe the form-meaning correspondence in section 3.3.

### 3.1. Semantic properties

In this subsection, I review a semantic property of the *because* X construction, comparing it with the more general *because*-clause constructions. Sweetser (1990) claims that a *because*-clause may be used in the content, epistemic, and speech-act domains, as exemplified in (3a-c):

(3) a. John came back because he loved her.
b. John loved her, because he came back.
c. What are you doing tonight, because there’s a good movie on. ( Sweetser 1990: 77)

Sentence (3a) describes a causal relation that holds in the real-world; that is, John loving her caused him to come back. In (3b), the causal relation is held in the epistemic domain; that is, the speaker’s knowledge about the fact that John came back causes him/her to conclude that John must love her. A speech-act *because*-clause as in (3c) serves as a motivation for performing a certain speech act such as asking about the interlocutor’s plans for the night.

Kanetani (2015, 2016, 2017, 2019) observes that the *because* X construction is skewed toward the content reading. This is confirmed by a survey the author conducted in January 2014 shortly after the American Dialect Society’s selection of (the innovative use of) *because* as its 2013 Word of the Year (see Kanetani 2015). Sentences (4a-e) below were developed for the survey and 24 native English speakers were asked about their acceptability. Of the 24 native speakers surveyed, seven speakers accepted the usage with different degrees of acceptability. The scores shown at the end of the examples are the average scores of acceptability by the seven respondents on a scale of 0 (unacceptable) to 3 (acceptable). The scores of those who did not accept the usage at all were eliminated from the calculations. ^2^

(4) a. He came back because love. (1.71/3.00)
b. I’m going to bed early because tired. (1.86/3.00)
c. He loved her, because back. (0.71/3.00)
d. [Looking at a wet ground] It’s rained, because ground. (0.00/3.00)
e. What do you wanna do on our first evening, because Paris? (0.57/3.00) ( Kanetani 2015: 66)

In (4a, b), the causal relations hold in Sweetser’s (1990) content domain. Sentences (4c, d) exemplify the epistemic *because* X, and sentence (4e) represents a speech-act *because* X. The results show that [ *because* X] appears to be acceptable in the content domain but not in the epistemic and speech-act domains.

This functional property might be predictable to some extent. Lakoff (1987) observes that a speech act construction that conveys a statement, like a rhetorical question, may occur in *because*-clauses when those clauses are in sentence-final position.

(5) a. We should go on a picnic, because isn’t it a beautiful day!
b. * Because isn’t it a beautiful day, we should go on a picnic. ( Lakoff 1987: 474)

The rhetorical question in the *because*-clauses in (5a, b), *isn’t it a beautiful day*, performs a state speech act conveying that it is a beautiful day. Hence, Lakoff calls these *because*-clauses performative subordinate clauses. As pointed out in Kanetani (2019), merely saying a sentence-final *because*-clause is not sufficient for a performative subordinate clause to occur.

(6) * He’s not going out for dinner because Japanese food, his wife is cooking. ( Kanetani 2019: 55)

Sentence (6) is ruled out even though the *because*-clause appears in the sentence-final position. In (6), the matrix negation scopes over the entire sentence, which is characteristic of the content *because*-clause (cf. Rutherford 1970). Thus, Kanetani (2019) concludes that it is epistemic/speech-act *because*-clauses that can be performative. As the sentence-initial position is reserved for content *because*-clauses (cf. Hirose 1999; Kanetani 2019), this generalization compensates for but is not incompatible with what Lakoff (1987) says.

As an epistemic/speech act *because*-clause performs a speech act of its own, the *because* X construction is naturally incompatible with an epistemic/speech-act reason clause, because the word or phrase that appears in the X-slot cannot perform an independent speech act. In this connection, *because of* NP (e.g., (1b)), one of the traditional uses, is also restricted to the content domain. An epistemic/speech-act *because*-clause is not replaceable with a *because of* phrase, as shown in (7):

(7) * He’s not coming to class, because of his having just called from San Diego. ( Rutherford 1970: 105)

In short, the use of [ *because of* NP] is restricted to the content domain for essentially the same reason as the [ *because* X] being limited to the content reading. That is, neither the NP that follows *because of* nor the word that directly follows *because* can perform an independent speech act.

Lastly, Okada’s (2020) discussion on the origin of the *because* X construction supports this claim. While acknowledging the difficulties in ascertaining when and how a new structure was generated, Okada (2020) considers the *because* X construction to have developed historically through the following steps. First, a blending occurs of *because* S and *because of* NP, yielding the new structure *because* NP, where the “NP works as a reference point for the conceptually relevant proposition” (Okada, p. 8). Subsequently, “the category restriction of the complement is nullified and elements of any category will appear as far as they work as reference points for the conceptually relevant proposition” (Okada, p. 8). ^3^ If this is correct, that is, if one of the inputs motivating the *because* X construction is the *because of* NP construction, it is not surprising that the meaning of the *because* X construction is skewed toward the content domain.

### 3.2. Formal properties

Let us turn to the formal properties of the *because* X construction. First, as a consequence of the functional properties observed in section 3.1, sentences with [ *because* X] behave in the same manner as those with a content *because*-clause. Both of them allow the reason part ([ *because* X] or the *because*-clause) to appear in sentence-initial position and to be focalized by an exclusive subjunct such as *only* and *simply.* ^4^ Relevant examples of the *because* X construction are given in (8a, b) and (9a, b).

(8) a. Because hurricane, the city is a mess. (1.71/3.00) ( Kanetani 2015: 68)
b. Because distance, since we know how fast light travels, if we know how far away a star is, we can also tell how old it is by knowing how long it would have taken to get there. (Corpus of Contemporary American English [COCA])
(9) a. Living people bother you because angry. Ghost make trouble only because sad, lost, contused. (COCA)
b. If a society needs a large, powerful law enforcement establishment, then there is something gravely wrong with that society; it must be subjecting people to severe pressures if so many refuse to follow the rules, or follow them only because forced. (Corpus of Global Web-Based English [GloWbE])

The acceptability score from participants was equally high for the constructed sentence in (8a) and was accepted by participants as highly as (4a, b). Sentence (8b) is an attested example from COCA. As mentioned in section 3.1, a sentence-initial *because*-clause is characteristic of the content reading. Hence, the acceptability of (8a, b) indicates that the *because* X sentences are compatible with the content reading. The [ *because* X] in (9a, b) is focalized by the exclusive *only.* As Kanetani (2019: chapter 4) claims, the focalization is possible for content *because*-clauses but not for epistemic/speech-act *because*-clauses. The ungrammaticality of (10) shows that the exclusive *just* cannot focalize an epistemic *because*-clause:

(10) * It has rained, just because the ground is wet. ( Kanetani 2019: 71)

Up to this point, it has been shown that the *because* X construction syntactically behaves like the content *because*-clause construction and not like the epistemic/speech-act *because*-clause constructions. 

To identify the formal property of the *because* X construction, it is necessary to consider what syntactic categories are likely to appear in the X-slot. The categorial restriction on the X-element is accounted for by the construction’s expressive structure to be proposed in section 4. Schnoebelen (2014) counts the target construction in tweets and groups all items that have 50 or more occurrences based on their parts of speech. The results are summarized in Table 1. Similarly, Bohmann (2016) examines 805 tweets and summarizes the categories that appear in the X-slot as in Table 2.

**Table 1.** Grammatical categories in the X-slot (based on Schnoebelen 2014).

Part of speech	Example	Rate (%)
noun	*people, spoilers*	32.02
compressed clause	*idc, ilism*	21.78
adjective	*ugly, tired*	16.04
interjection	*sweg, omg*	14.71
agreement	*yeah, no*	12.97
pronoun	*you, me*	2.45

**Table 2.** Grammatical categories in the X-slot (based on Bohmann 2016: 161).

Part of speech	Rate (%)
Noun/NP	38.8
Interjection	20.3
Reduced clause	14.5
Adjective	9.8
Other	16.6

The two tables commonly include nouns (or noun phrases), adjectives, and interjections. The most recent corpus survey by Mendes Junior and Mattos (2021) also confirms that the categories occurring frequently in the X-slot are (in descending order) nouns > adjectives > interjections > adverbs > verbs. Thus, it is safe to say that nouns, adjectives, and interjections frequently appear in the X-slot.

In addition to these three categories, several other categories are identified. First, in comparing Tables 1 and 2, it should be noted that Schnoebelen’s (2014) “compressed clause” is not the same as Bohmann’s (2016) “reduced clause”. In Schnoebelen’s survey of tweets, the most frequent token is *yolo*, which is a compression of *you only live once.* Schnoebelen states that “if you spell it out, *because you only live once* is actually completely standard ( *you only live once* is an example of a fine full clause). But *yolo* is a lot like an interjection” (underline added). Bohmann (2016: 161) also distinguishes these compressed clauses from their clausal counterparts and considers these “(semi-)lexicalized, fixed expressions”. As special forms generally convey special functions, following Schnoebelen (2014) and Bohmann (2016), I take compressed clauses as fixed expressions with a function similar to that of interjections. ^5^

As noted above, the reduced clause in Table 2 is distinguished from the compressed clause in Table 1. Bohmann (2016) defines reduced clauses as finite clauses “often with deleted subjects” (p. 160). There is one thing we must bear in mind in dealing with reduced clauses. Namely, in some cases, a reduced clause follows *because* while the sentence does not exemplify the *because* X construction. As Okada (2020) points out, subordinate clauses generally allow the subject and copula to be deleted. Observe (11):

(11) a. This would at least be honest, though I think it would be unwise, because unnecessary. BETTER TO GIVE EVERYBODY A FAIR CHANCE. (Corpus of Historical American English [COHA] 1820)
b. And a Bostonian, appeals to history, and shows that Boston is first, because oldest. (COHA 1823)
  (cited from Bergs 2018: 45)

Using these examples, Bergs (2018) argues that the alleged new usage was attested as early as the early 19th century. Okada (2020) critically examines these sentences and points out that “considering [(12)], the examples in [(11)] do not appear at all innovative. Rather, they only conform to the regular deletion process observed widely in subordinate clauses” (p. 9).

(12) a. Although no longer a minister, she continued to exercise great power.
b. While in Paris, I visited Uncle Leonard.
  ( Huddleston and Pullum 2002: 1267)

That is, Okada claims that the sentences in (11a, b) do not exemplify what we call the *because* X construction because their (superficial) *because* X part may be recoverable based on the matrix subject being combined with a copula verb. Incidentally, based on a survey of the *Oxford English Dictionary*, Okada (2020) reports that this type of “subject + copula” deletion in a *because*-clause dates as far back as the 16th century. Therefore, the systematic “subject + copula” deletion structure should be distinguished from Bohmann’s (2016) reduced clause and eliminated from the analysis (at least for the present purposes); thus, the “reduced clause” is limited to examples such as the following:

(13) Bye going to study for English because didn’t finish this morning because fell asleep.
  ( Carey 2013, underlines added)

Lastly, “agreement” and “pronoun” are taken as independent categories in Table 1. Bohmann might include them (if at all) in the “other” category. It is necessary to highlight the relatively small number of words in these categories. The agreement words include *yes*, *yeah*, *no*, and a few other similar words; the pronoun is a closed category consisting of only a small number of members. Nevertheless, agreement words appear far more frequently than pronouns in this construction. In Table 1, “agreement” (12.97%) actually nears “interjection” (14.71%), one of the most frequently used categories. By contrast, pronouns (2.45%) are used far less frequently. This is also supported by McCulloch (2014), who observes that a pronoun is “weird” when used in this construction.

(14) ?? I can’t go to the party because you. ( McCulloch 2014)

In short, the pronoun is a marginal (if not impossible) category as an X-element.

Thus, nouns, adjectives, interjections, and agreement words are frequently used in this construction. Compressed clauses are analyzed in a parallel fashion to interjections ( Schnoebelen 2014; Bohmann 2016). Reduced clauses also need to be considered. Mendes Junior and Mattos (2021) observe that verbs and adverbs can be used at low frequencies (cf. also Okada 2020). However, pronouns are not used ( McCulloch 2014) or are rare ( Schnoebelen 2014). Mendes Junior and Mattos (2021: 37) highlight the incompatibility of function words with the construction:

“[D] evido à brevidade típica de [ *because X*], o item lexical que preenche a posição [X] deve ser semanticamente relevante e pertinente ao conteúdo introduzido no enunciado antes do *because.* Parece ser por esse motivo pelo qual palavras funcionais sofrem restrição em [X].”

(Because of the typical briefness of [ *because* X], the lexical item that fills the position [X] must be semantically relevant and pertinent to the content introduced in the statement before the word *because.* This seems to be why function words are restricted in [X].)

(author’s translation and underlines added)

The mechanism by which to account for the (non-)occurrence of these elements will be further discussed in section 4.

## 3. Form-meaning pairing of the *because* X construction

From the observations given so far, the form-meaning pairing of the *because* X construction may be described as in (15):

(15) [CLAUSE _i_ *because* X _j_] ↔ [P (evoked by “X _j_”) is a reason for Q _i_]
  (Modified from Kanetani 2017: 95)

In (15), the form of the construction specified on the left side of the double-headed arrow (↔) is paired with the meaning specified on the right side; the coindexed elements in the form-pole and meaning-pole represent the form-meaning correspondences. That is, CLAUSE _i_ conveys the propositional meaning Q _i_; X _j_ with the meaning represented as “X _j_” is a word (or phrase) from one of various categories such as those listed in Tables 1 and 2. Since a *because* X sentence represents a real-world causal relation in the content domain (section 3.1), the X-element needs to represent a certain propositional content. Therefore, the meaning of the word in the X-slot cannot be taken as a simple denotation of the lexical/phrasal meaning but should be understood as a relevant proposition evoked by it. ^6^

Note that the form-meaning pairing in (15) is a base-level representation and that there are variations. For example, [ *because* X] may precede the main clause (e.g., (8a, b)); there are also cases—as used in colloquial or online contexts—where the main clause is reduced or omitted (e.g., *Early morning gym because fat* ( Bohmann 2016: 149)), as well as cases where orthographic variations of *because* (e.g., *bc*, *cuz*, and *coz*) are involved. However, differences between these formal variations are not considered in the present article.

## 4. Two-layered expressive structure: Private expression within public expression

In section 3.2, I observed that nouns, adjectives, interjections (including compressed clause), agreement words, and reduced clauses frequently appear in the X-slot. To account for their frequent appearances in the X-slot, in this section, I investigate the construction’s expressive structure in Hirose’s (2000) terms: “private expression” and “public expression”. Specifically, following Kanetani (2016, 2017, 2019), I claim that the element in the X-slot serves as a private expression. Hirose (2000: 1624) proposes two levels of linguistic expressions, called *private* and *public expression*: the former is “the level of linguistic expression corresponding to the non-communicative, thought-expressing function of language”, whereas the latter is “the level of linguistic expression corresponding to the communicative function of language”. Thus, the claim being made here may be rephrased as follows: the element in the X-slot has a thought-expressing function with no intention of communication.

However, I do not claim that the whole construction functions as a private expression. Schnoebelen (2014) reports that 36% of the tweets investigated involve @-mentions, which indicates that they are aimed at a specific person or persons as a reply (cf. also Bohmann 2016), and therefore that the *because* X construction seems skewed toward the “interpersonal”. Thus, the expressive structure of the construction may be illustrated as in (16), with Hirose’s (2000) notations of private expression represented in angle brackets with the subscript “Priv” < _Priv_...> and public expression represented in square brackets with the subscript “Pub” [ _Pub_ ...].

(16) [ _Pub_ *because* < _Priv_ X>]

The representation in (16) indicates that the whole message is communicated as a public expression, within which a private expression is encapsulated. With this structure in mind, in the following subsections, I examine the “privateness” of the expressions that frequently appear in the X-slot and the “publicness” of those that rarely appear.

### 4.1. Interjections and compressed clauses

Interjections frequently appear in the X-slot as in (17):

(17) a. That feeling you get when you finish an essay and you just want to cry because yay.
b. Admittedly, not in the UK yet, because aargh.
  ( Carey 2013)

Interjections are described as “purely emotive words” ( Quirk *et al.* 1985: 853) that “have expressive rather than propositional meanings” ( Huddleston and Pullum 2002: 1361); that is, they are used to express, rather than to communicate, the speaker’s emotion. Therefore, interjections by nature may serve as private expressions with no intention of communication.

How then is the conveyed message understood by the hearer? Padilla Cruz (2009) examines cases where a subordinate clause is replaced by an interjection as in (18):

(18) She is so beautiful that ... oh! ( Padilla Cruz 2009: 190)

He explains that “the hearer could recover the missing clause using contextual and/or encyclopedic information” ( Padilla Cruz 2009, pp. 190-191) and that the meaning of sentence (18) may be understood as something in (19a-c) or the like:

(19) a. She is so beautiful that I like/love her.
b. She is so beautiful that I have fallen in love with her.
c. She is so beautiful that I would very much like to marry her.
  ( Padilla Cruz 2009: 190)

It is important to note that specific emotions are mapped onto each interjection; for example, *aargh* is used to express “fear, anger, or other strong emotion” ( *Oxford Advanced Learner’s Dictionary*, 8th edition [OALD ^8^]), and *yay* is used to show that one is “very pleased with something” (OALD ^8^). Thus, the utterance *because aargh* may be construed as *because something extremely bad happened.* Therefore, the lexical information as well as the “contextual and/or encyclopedic information” plays an important role in recovering the message.

My earlier analysis fails to distinguish the roles of a speaker and hearer, and only identifies a metonymic relation between the semantic content of an interjection and that of a clause ( Kanetani 2015). However, by using an interjection, the speaker merely expresses an emotion with no intention of communicating; it is the hearer who attempts to understand the utterance in question based on the contextual, encyclopedic, and/or lexical information (cf. Padilla Cruz 2009; Kanetani 2016).

As observed in section 3.2, compressed clauses such as *yolo* and *ilysm* (a compression of *I love you so much*) have a similar function to that of interjections. In fact, they exhibit certain features distinct from their clausal counterparts. For instance, the compressed clause *yolo* is pronounced in an exclamatory tone, conveys specialized meanings, and can be converted into the verb *yoloing* ( https://www.youtube.com/watch?v=MKT3DaClfvY [retrieved on July 2, 2021]; see also Bohmann 2016: 161), which are shared features with interjections and not with finite clauses. Because of the meaning of the clausal counterparts (e.g., *yolo* for *you only live once*), compressed clauses may convey more specific meanings close to clauses than simple interjections. In the present article, however, compressed clauses used in this construction are profitably analyzed in a similar way to interjections (cf. Schnoebelen 2014, Bohmann 2016). ^7^

### 4.2. Content words

Content words include nouns, adjectives, verbs, and adverbs, constituting the proposition being conveyed. However, it is the hearer who recovers the proposition containing these words as its parts, with the aid of the PART FOR ALL—more specifically, WORD FOR CLAUSE—metonymy. The speaker, on the other hand, simply chooses the most salient word from a clause conveying the meaning of “P” in (15) as a reason (see also footnote 6). What is “salient” may be something that pops into the speaker’s mind at the time of utterance; therefore, the word represents the speaker’s private expression to the extent that he/she does not need to place others at the center of his/her consciousness. Nouns and adjectives are open-set content words whose primary function is “to carry the meaning of a sentence” and hence “typically carry the burden of the semantic content of utterances” ( Cruse 2011: 267f.). Apart from nouns and adjectives, adverbs and verbs are also open-set content words that can be used in this construction ( Okada 2020; Mendes Junior and Mattos 2021). The use of verbs and adverbs in this construction may also be accounted for on the same ground as the use of nouns and adjectives.

Recall that pronouns are not used in this construction, as observed by McCulloch (2014):

(20) ?? I can’t go to the party because you. (= (14))

This observation is compatible with the present proposal that the X-element represents the speaker’s private expression. In terms of Hirose’s (2000) dichotomy between private and public expressions, English personal pronouns are primarily defined as public expressions that can be diverted to represent the private self (cf. Hirose 2000, 2015). It is worth quoting Benveniste (1971: 224f.) here:

“[C] consciousness of self is only possible when it is experienced by contrast. I use *I* when I am speaking to someone who will be a *you* in my address. It is this condition of dialogue that is constitutive of person, for it implies that reciprocally *I* becomes *you* in the address of the one who in his turn designates himself as *I.*” (underline added)

In short, only relatively to others can the personal pronoun be defined and used; that is, pronouns cannot be used in the absence of others. ^8^ This makes personal pronouns unsuitable X-elements, because the slot requires a private expression.

McCulloch (2012) proposes another intriguing restriction on the nominal category. She observes that the noun that follows *because* should be a bare noun, i.e., a noun with no determiner, as in (21). Bergs (2018: 49) also reports that “all examples in COCA and COHA have bare nouns” and observes that adding a prenominal modifier or determiner diminishes the acceptability, as shown in (22).

(21) * I can’t come out tonight because essay [sic.]/my essay/an essay/this essay. ^9^ ( McCulloch 2012)

(22) [...] “Because (?favorable/?the) circumstances. I was just lucky, really …”
  ( Bergs 2018: 49, based on COCA)

This restriction also indicates the private nature of the X-element. According to Quirk *et al.* (1985: 253), “when used in discourse, noun phrases refer to the linguistic or situational context. The kind of reference a particular noun phrase has depends on its determinative element, i.e. the item which ‘determines’ it”. In other words, determination is necessary in a discourse for the speaker to allow the hearer to identify the type of reference. Put differently, unless the speaker has an interlocutor in mind, determination is not necessary in Quirk *et al.*’s sense.

So far, two restrictions on the nominal category that indicate the privateness of the X-element have been discussed. While other content words may be analyzed in the same way as nouns because they are subjectively selected as possible salient constituents of the corresponding clause, an additional comment is needed on verbs, which are only rarely used in this construction. An example with a verb is given in (23):

(23) Set an alarm for 8 so I could get up and be productive early. Reset an alarm for 930 because sleep.
  ( Schnoebelen 2014)

According to Schnoebelen (2014), verbs frequently used in this construction, *stop*, *want*, and *sleep*, may be considered nominal expressions. The word *sleep* in (23) may be a bare noun, as Schnoebelen suggests, but it can be analyzed as a verb. Notice that the verb *sleep* appears here in its bare form. If used in a canonical *because*-clause, the verb should inflect for the past tense, as shown in (24): ^10^

(24) I set an alarm for 8 so I could get up and be productive early. I reset an alarm for 930 *because I slept again.*

The fact that the verb in (23) appears in the bare form is parallel to the fact that bare nouns are preferred in this construction ( McCulloch 2012; Bergs 2018). ^11^

In summary, the restrictions on nouns and verbs may be reduced to the lack of what generative linguists call functional categories corresponding to the D- and T-heads, respectively. Konno (2012) points out that the lack of a functional category is related to the lack of hearer-orientedness (cf. also Konno 2015). For example, a Mad Magazine sentence (e.g., *Him wear a tuxedo?!*) that is used to “express surprise, disbelief, skepticism, scorn, and so on, at some situation or event” ( Akmajian 1984: 2) cannot be embedded in a verb of commutating such as *tell* (e.g., *??Mary told him “Him wear a tuxedo?!”* ( Konno 2015: 146)) *.* Konno thus views the construction as having an exclusively private function. Notably, the verb in a Mad Magazine sentence is bare and hence lacks tense. Following Konno (2012, 2015), we may posit that the bare nouns used in the *because* X construction and the bare verb *sleep* in (23) exhibit the speaker’s private expression. ^12^

### 4.3. Agreement words

This subsection considers agreement words, as in (25):

(25) “So I guess you’re okay that it’s you then?” he says, and Nick grins because yeah. “Very okay.” (GLoWbE)

In (25), *because* is followed by *yeah*, an agreement word, in which the speaker asserts only the polarity of the propositional content with the other details being underspecified. The word *yeah* in (25) affirms the proposition that he is okay. In this way, agreement words such as *yes* or *yeah* affirm certain propositions that lie behind the words, while disagreement words such as *no* deny them. To maintain this claim, let us observe Nakau’s (1994) hierarchical structure of a proposition, as illustrated here (26): ^13^

(26) [ _PROP4_ POL [ _PROP3_ TNS [ _PROP2_ ASP [ _PROP1_ PRED (ARG _1_, ARG _2_,....ARG _n_)]]]]
  (adapted from Nakau 1994: 15)

As shown in (26), a full proposition consists of the four strata PROP1-PROP4. The lowest layer, PROP1, consists only of the combination of the predicate and its argument(s). Added over PROP1 are the aspectual, tense, and polarity operators, yielding more complex and composite propositions. As the polarity operator is placed at the outermost layer in (26), the proposition that exists behind the agreement words corresponds to PROP4. As with the interjections, the hearer may recover the missing part, PROP3 in this case, by using contextual and/or encyclopedic information.

Framed in Nakau’s (1994) model (26), the content words, such as nouns and verbs, used in the bare form correspond to part of PROP1, either PRED or an ARG, with no tense or aspectual operator attached. Crucially, either a content word as part of PROP1 or an agreement word as part of PROP4 may serve as a reference point to evoke a full proposition (for the use of Langacker’s (1993) term *reference point*, see Okada 2020). Generally, when both the speaker and hearer are assumed to be cooperative, the speaker should make his/her contribution as informative as is required ( Grice 1975). The use of a private expression, however, does not assume the existence of a hearer, and hence the speaker can express a situation as he/she construes it.

Therefore, the X-elements observed in sections 4.1-4.3 are representations of the speaker’s private expressions. Interjections merely reflect how the speaker takes a certain situation; content words typically appearing in the bare form and agreement words refer to part of a proposition that the speaker constructs in response to the situation construal. Thus, the speaker encapsulates these elements in the X-slot and leaves the remainder of the relevant propositional content unspecified.

## 4.4. Reduced clauses

This subsection examines reduced clauses, which indicate privateness in a different way from the other cases observed in sections 4.1-4.3, as they are a clausal category while the others are lexical categories. Following Bohmann’s (2016: 160) definition, I take reduced clauses as finite clauses with deleted subjects, as shown in (27):

(27) Bye going to study for English because didn’t finish this morning because fell asleep. (= (13))

In (27), the subject pronoun *I* is omitted. To deal with reduced clauses of this kind, Hirose and Hasegawa’s (2010) analysis of diary English is helpful. They observe that reduced clauses (or “null subject sentences” in their terms) are commonly found in diaries (cf. Haegeman and Ihsane 1999). They cite the following examples from Helen Fielding’s diary-style novel *Bridget Jones’s Diary*: ^14^

(28) () Was just leaving flat for work when () noticed there was a pink envelope on the table …
  ( Hirose and Hasegawa 2010: 63)

In (28), the subject *I* is omitted both in the matrix clause and in the adverbial clause. Hirose and Hasegawa (2010: 67) account for the distribution of this construction as follows. As we will see in detail in section 5, English is by default a public-self-centered, other-oriented (hence, highly objective) language. However, when used in a special context like a diary where communication is not intended, the language need not linguistically encode what the speaker presupposes about him- or herself, exhibiting self-orientedness (or high subjectivity). In this sense, the reduced clauses in (28) may be regarded as representations of private expressions that are restricted to specific registers such as a diary, which is not aimed at a hearer/reader. Likewise, the reduced clauses that follow *because* in (27) may be considered private expressions. ^15^

## 5. The three-tier model of language use

In section 4, I claimed that the element in the X-slot serves as a private expression, thereby proposing the two-layered expressive structure as in (29):

(29) [ _Pub_ *because* < _Priv_ X>] (= (16))

Crucially, the element in the X-slot serves as a private expression. This section considers what it means that a private expression is used in a public expression in terms of the three-tier model of language use ( Hirose 2015).

As mentioned in section 2, the three-tier model is a grammatico-pragmatic theory proposed *inter alia* by Hirose (2015) as a natural extension from the deconstruction of the speaker into the private self as the subject of thinking and the public self as the subject of communicating ( Hirose 2000). According to the three-tier model, language use comprises the three tiers listed in (30a-c), and “languages differ as to how the three tiers are combined, according to whether their basic egocentricity lies in the public self or the private self” ( Hirose 2015: 123).

(30) a. *situation construal tier*: the speaker as private self construes a situation, forming a thought about it.
b. *situation report tier*: the speaker as public self reports or communicates his construed situation to the addressee.
c. *interpersonal relationship tier*: the speaker as public self construes and considers his interpersonal relationship with the addressee.
  (ibid.: 123)

In this model, the English language is characterized as follows:

“In English, a public-self-centered language, the situation construal tier is normally unified with the situation report tier, to which is added the interpersonal relationship tier […]. The unification of situation construal and situation report means that one gives priority to the outside perspective from which to report a situation and linguistically encodes as much as is necessary to do so. Thus, even when the speaker himself is involved in a situation as a participant, the reporter’s perspective places his self as a participant on a par with the other participants; hence comes objective construal. On the other hand, the fact that the situation report tier is not unified with the interpersonal relationship tier means that one can assume an unmarked (or neutral) level of communication which does not depend on any particular relationship between speaker and addressee, a level where the speaker and the addressee are assumed to be linguistically equal, being in a symmetrical relationship.” (ibid.: 123-124, underlines added)

To highlight the characteristics of English, let us compare them with those of Japanese, which is described as follows:

“In Japanese, a private-self-centered language, the situation construal tier is normally independent of the situation report tier and the interpersonal relationship tier […]. Thus, in construing a situation, the speaker can freely place himself in the situation and view it from the inside; also, he does not need to linguistically encode what is already given in his consciousness; hence comes subjective construal. On the other hand, the situation report tier is unified with the interpersonal relationship tier, which means that in reporting a situation to someone, the speaker must always construe and consider his relationship with the addressee, defining himself and the addressee in terms of that relationship. Thus, in situation report, interpersonal relationship is linguistically encoded as much as possible, and there is no unmarked level of communication neutral to interpersonal relationship.” (ibid.: 124-125, underline added)

**Figure 1.**
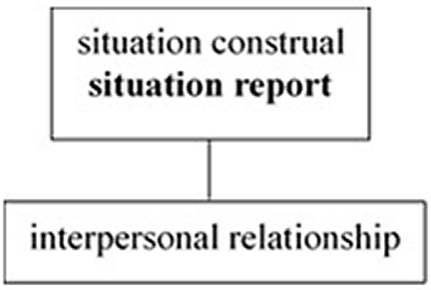
English as a public-self-centered language.

**Figure 2.**
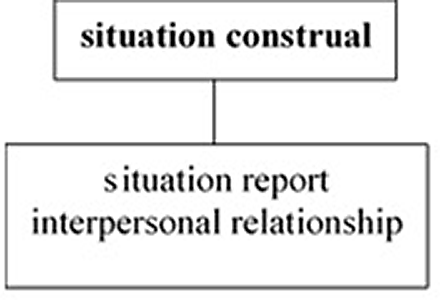
Japanese as a private-self-centered language.

The crosslinguistic difference in the unification pattern of the three tiers is illustrated in Figures 1 and 2, where the bold faces indicate the tiers in which the unmarked deictic center is located, i.e., the default position where deictic expressions are interpreted. ^16^

Figure 1 illustrates that the situation report tier, where the unmarked deictic center is located, is unified with the situation construal tier in English. Therefore, English speakers need to construe the situation objectively as they report it to others. Conversely, Figure 2 shows that, in Japanese the unmarked deictic center is located in the situation construal tier, which is independent of the unification of the situation report and interpersonal relationship tiers, allowing Japanese speakers to express the situation as they construe it.

**Figure 3.**
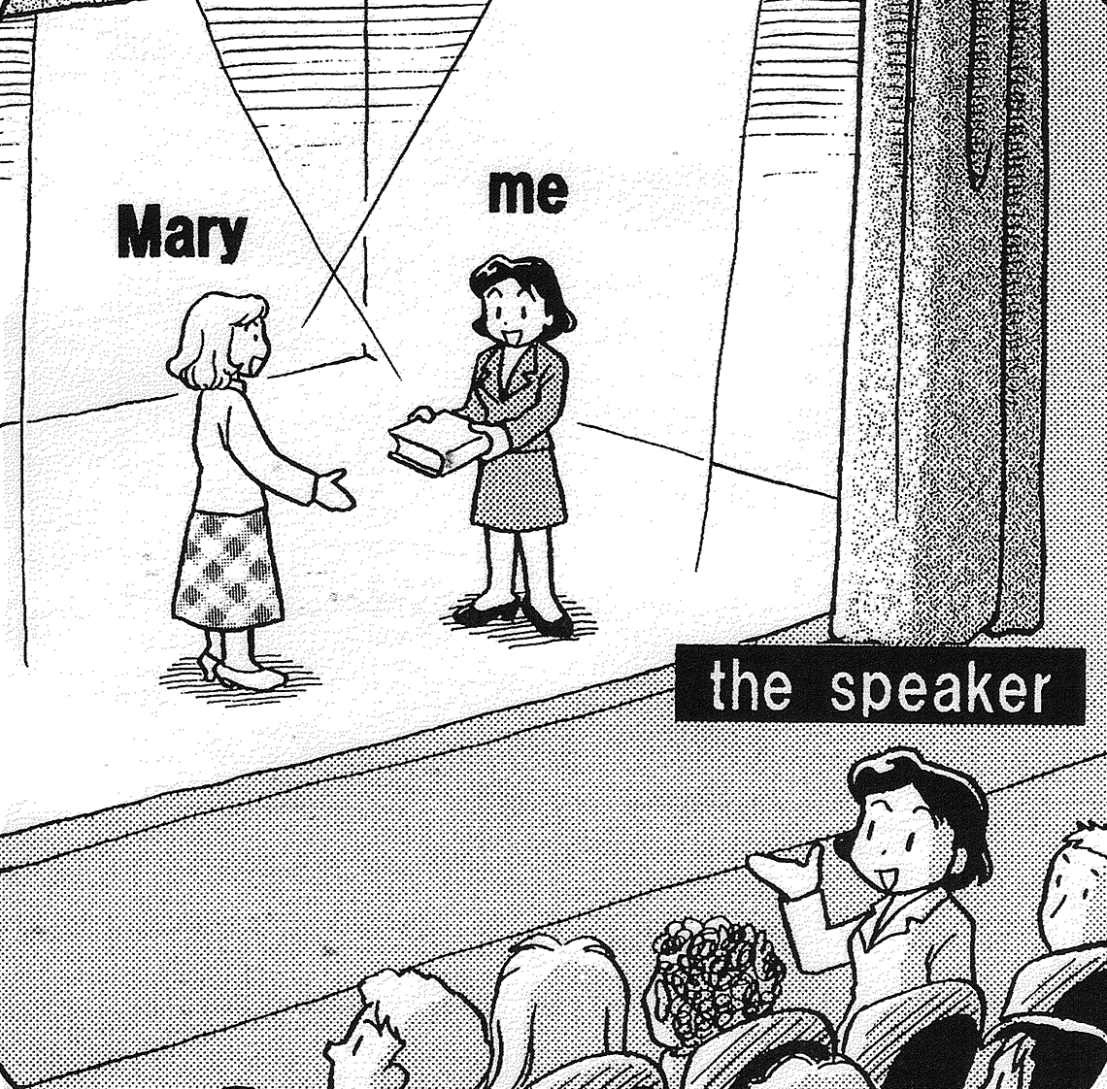
*Mary gave me this book* [English] ( Ide 2006: 223).

**Figure 4.**
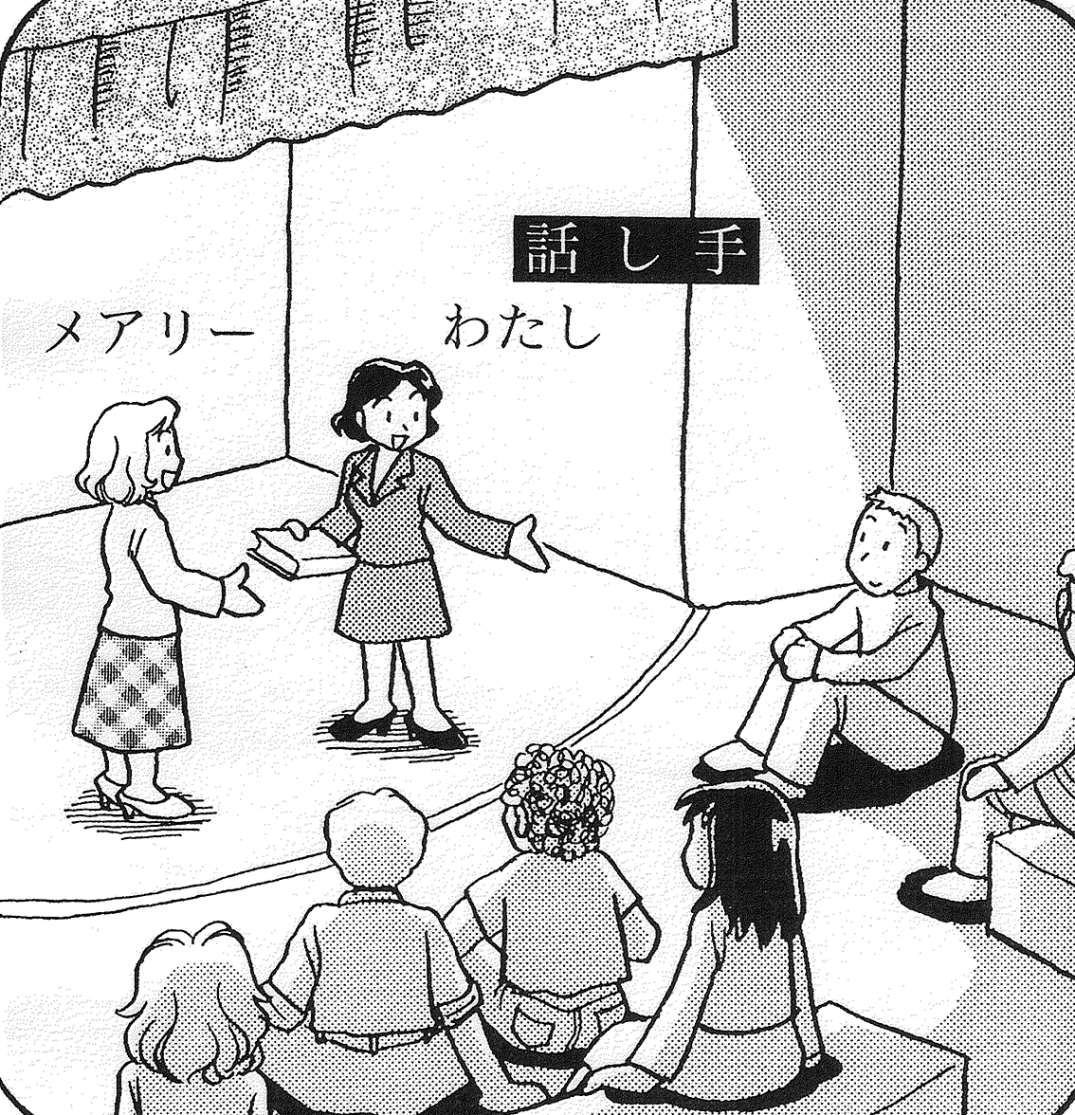
*Mary gave (me this book)* [Japanese] ( Ide 2006: 222). ^19^

Ide (2006) also neatly describes the typological difference. According to Ide, while Japanese speakers tend to view themselves as participants in the situation described, English speakers tend to take the perspective of an omniscient narrator and overview the entire speech event from the outside. The different perspectives of the two languages are illustrated in Figures 3 and 4. ^17^

As illustrated in Figure 3, an English speaker sees and describes herself on stage as others see her. A Japanese speaker, illustrated in Figure 4, plays the role of a participant on stage as well as the narrator. The speaker, who is embedded in the situation, has to linguistically encode only what is necessary and leaves other elements ( *me*, *this book*) unspoken. Thus, to describe the same situation, the Japanese speaker would say as follows, with the parenthetical elements not necessarily being expressed. ^18^

(31) Mearii-ga  (kono hon-o watashi-ni) kureta-noyo
  Mary-Nom  (this book-Acc 1.Sg.-Dat) gave-SPF
  ‘Mary gave (me this book).’

In short, English speakers prefer to take an objective perspective from outside of the situation while Japanese speakers prefer to take a subjective perspective from the inside. This observation is compatible with the three-tier model.

Another important typological difference Hirose (2015) puts forward as a consequence of the three-tier model is that the unmarked mode of expression is public expression in English and private expression in Japanese. Consider the following contrast:

(32) Kyou-wa  doyoubi da.
  today-Top Saturday Cop
  ‘Today is Saturday.’

(33) Kyou-wa doyoubi {da yo/  desu/  degozaimasu}.
  today-Top Saturday {Cop SFP/  Cop.Pol/ Cop.Super-Pol}
  ‘Today is Saturday.’
  ( Hirose 2015: 122)

The unification of the situation construal and situation report tiers in English means that the utterance *today is Saturday* has a performative structure (cf. Ross 1970), as in (34):

(34) I SAY TO YOU today is Saturday
     ↑       ↑
  situation report situation construal
  (adapted from Hirose 2015: 128)

Thus, the unmarked mode of expression in English is considered public expression. In contrast, the Japanese unmarked sentence in (32) cannot have a similar structure to (34), as shown in (35):

(35) # I SAY TO YOU kyoo-wa doyoobi-da ( Hirose 2015: 128)

Note that all of the Japanese sentences in (32) and (33) convey one and the same propositional content, *today is Saturday.* The unmarked sentence in (32), however, functions as a private expression by itself; hence, it is incompatible with the performative clause, as shown in (35). Instead, various expressions sensitive to the interpersonal relationship, such as the unmarked sentence-final particle *yo*, the (super) polite form of the copula *desu* or *degozaimasu*, etc., are employed to make the expression public.

With the typological characteristics of English in mind, let us consider the fact that the X-element represents the speaker’s private expression. According to the three-tier model, an English speaker essentially takes a reporter’s (or an objective) perspective and linguistically encodes as much as is necessary to do so, which makes the unmarked mode of expression in English public expression. The expressions in the X-slot, on the other hand, exhibit the speaker’s subjective construal in that the speaker does not linguistically encode what is already given in his/her consciousness, which is characteristic to languages like Japanese. This claim is in line with Bergs’s (2018) argument that the *because* X construction is subjective compared with the content causal *because*-clause construction in the sense of Traugott and Dasher (2004). Crucially, it is not the whole construction but only its part that deviates from the norm of the English language. Presumably, the subjectivity Bergs observes in this construction is related to the subjective nature of the X-element. In the following sections, I examine the subjectivity of the X-element.

## 6. Privateness and content causal relation

In section 3, I claimed that the meaning of the *because* X construction is restricted to Sweetser’s (1990) content causal relation. After observing in section 4 that the X-element serves as private expression, I pointed out in section 5 that the X-element exhibits a characteristic of a private-self-centered language like Japanese. In this section, I consider how these facts are intertwined along with Kanetani’s (2017) view of a speech act unit as a small discourse.

While Sweetser (1990) proposes the three domains in which *because* functions, Kanetani (2019) claims that the epistemic and speech act *because*-clauses should be grouped together and that the distinction of the content causal relation from epistemic/speech-act causal relation is crucial. The division is based on how speech act units are formed. A sentence in the content domain performs one speech act as a whole, whereas two independent speech acts are performed in the epistemic/speech-act domain. Compare the following sentences, where the arrows (↑ and ↓) indicate intonation patterns:

(36) a. Is the ground wet because it has rained?↑ ( Kanetani 2019: 46)
b. Has it rained,↑ because the ground is wet.↓ (ibid.: 54)

Sentences (36a, b) are interrogative sentences with a content *because*-clause and an epistemic *because*-clause, respectively. The rising intonation appears at the end of the sentence in (36a), which indicates that the scope of the question encompasses the whole sentence, thus performing a single speech act: The rising intonation in (36b) appears at the end of the main clause. That is, the question scopes over the main clause, while the *because*-clause independently performs a statement speech act (see section 3.1; cf. also Lakoff 1987). Thus, the distinction of the epistemic and speech act *because*-clauses may be reduced to the kind of speech act (e.g., a statement, question, or imperative) being performed in the main clause ( Kanetani 2019).

Given the differences in speech act unit formation between the content *because*-clause construction and the epistemic/speech-act *because*-clause construction, I recast each speech-act unit here as a “small discourse” to account for the relation between the content reading and the private nature of the X-element in the *because* X construction. Namely, the content *because*-clause construction consisting of one speech act unit can be taken as a discourse, which starts with the situation described in the main clause and ends with the situation described in the *because*-clause. By contrast, in the epistemic/speech-act *because*-clause construction, there exist two paratactic discourses that are independent of each other. For instance, the content causal sentence *the ground is wet because it has rained* depicts (as it were) one scene, whereas the epistemic causal sentence *it has rained, because the ground is wet* depicts two separate scenes, one about raining and the other about the ground being wet. The obligatory comma intonation between the main clause and the epistemic/speech-act *because*-clause (Sweetser 1990) symbolically represents the discourse boundary or the scene shift.

Together with the notion of small discourse, let us consider the *because* X sentence in (2), repeated here as (37):

(37) I cannot go out with you today because homework/sick. (= (2))

Since the sentence describes a content causal relation, its discourse structure is also assumed to be the same as that of the content *because*-clause construction. Specifically, the sentence delivers a discourse on the speaker being unable to go out because of his/her homework/sickness. At the beginning of the discourse, the speaker takes the reporter’s perspective, placing his/herself in a situation as a participant, just as with the speaker in Figure 3. That is, the speaker starts the discourse with the unmarked mode of expression in English, playing the role of a narrator, who observes the situation objectively standing on a par with the hearer. As the discourse progresses, however, the speaker switches his/her perspective to a perspective from the inside, describing the situation as a participant, as if he/she jumped into the situation and fused with his/herself on stage, just like the speaker illustrated in Figure 4.

What then makes the speaker switch perspectives? As seen in section 4, the *because* X construction itself has a public function ( Schnoebelen 2014; Bohmann 2016). Therefore, by starting with the unmarked mode of expression in English, the speaker indicates the publicness of the expression while avoiding the abrupt occurrence of a private expression. Then comes a private expression which indicates his/her own thought expression. As such, it naturally follows that the epistemic/speech-act causal relations are restricted *.* As mentioned earlier in this section, an epistemic/speech-act *because*-clause introduces a new discourse independent of the main clause, even though the *because*-clause appears after the main clause. Thus, the speaker needs to start the new discourse in the unmarked mode.

Some examples may seem problematic for the small-discursive account. Consider the following examples:

(38) a. NSF cancels new political science grants because … politics. (Twitter)
b. Because distance, since we know how fast light travels, if we know how far away a star is, we can also tell how old it is by knowing how long it would have taken to get there. (= (8b))

In (38a), the main clause subject, the NSF (National Science Foundation), is a third person and is not identical with the speaker who jumps into the situation toward the end of the discourse. In (38b), on the other hand, the [ *because* X] part appears sentence-initially, so it seems difficult to maintain the idea that the speaker switches his/her perspective as the discourse progresses.

Let us first consider example (38a). The actual tweet is linked to a blog written by the same person, where a detailed explanation is given, as in (39):

(39) A couple of weeks before the deadline for new grant proposals in political science were due, the NSF has canceled the program, at least for this grant cycle. No explicit reason was given, but everyone knows why it happened. Back in March, Congress passed the Coburn Amendment to the Continuing Appropriations Act of 2013, which limits political science funding to research that “promotes national security or the economic interests of the United States.” … .
  ( http://www.preposterousuniverse.com/blog/2013/08/05/national-science-foundation-cancels-call-for-new-political-science-grant-proposals/ [retrieved on July 2, 2021, underlines added])

The first section underlined in (39) “the NSF has canceled the program, at least for this grant cycle” makes virtually the same statement as the main clause in (38a). The other underlined part states the reason: “Congress passed the Coburn Amendment to the Continuing Appropriations Act of 2013, which limits political science funding to research that ‘promotes national security or the economic interests of the United States’”. However, as is clear from the sentence between the two underlined parts, the reason is the author’s opinion. In short, in (38a), using the word *politics*, the author represents the NSF’s intention and explains it from his own point of view. Thus, in (38a), the author starts the discourse with the narrator’s perspective and then presents his own private expression while maintaining the narrator’s perspective without being fused with any participant in the situation.

The other case we need to consider is (38b), where the [ *because* X] precedes the main clause. When a *because*-clause appears in sentence-initial position, it is contextually presupposed. To confirm this, consider the following dialogue:

(40) A: Why is the ground wet?
  B: #Because it has rained, the ground is wet.

In (40), speaker B’s response to A’s question is anomalous. The response should assert the reason for the ground being wet; nevertheless, the sentence-initial *because*-clause indicates that the reason is contextually presupposed. By the same token, sentence-initial [ *because* X] may be considered to be contextually presupposed. In other words, a sentence-initial [ *because* X] may be used only within a context where X is established as (part of) a topic. This can be confirmed by seeing the actual context of use, as COCA allows us to check the context in which the sentence is used. Sentence (38b) appears during an interview on the performance of a space telescope, where the interviewee talks about how distant galaxies can be resolved into individual stars with the telescope. To this extent, sentence (38b) causes no abruption.

## 7. Significance of the X-element serving as private expression

Thus far, I have claimed that the *because* X construction has a two-layered expressive structure without considering its motivations. In this section, I examine the meaning of the two-layered structure from metapragmatic strategy perspectives. Some phenomena with the two-layered expressive structure in Japanese have been reported in the literature. First, Naya (2017) investigates an innovative use of *kudasai* ‘please’ in social networking services, as in (41), which is distinguished from its canonical use with the *te*-conjunctive form *ore-no tooan-o tensakushite kudasai* ‘please correct my answer(s)’.

(41) Ore-no touan-o tensakushiro kudasai
  1. Sg-Gen answer-Acc correct. Imp please
  ‘Please correct my answer(s).’
  ( Naya 2017: 63)

Naya regards the imperative form *tensakushiro* ‘correct _IMP_’ as bearing a private function, which is turned into a public expression by adding *kudasai* ‘please’. That is, the sentence is interpreted as expressing dual messages, as in (42): the speaker’s wish as expressed by the private expression and the speaker’s request as expressed by the public expression.

(42) I say to you that I strongly wish someone to correct my answers. ( Naya 2017: 74)

Since the construction is used exclusively in the environment of online communication, Naya argues that its use is motivated by this environment, in which some can fulfill the speaker’s wish but others cannot. Those who can correct the speaker’s answers may understand the sentence as an indirect request, while those who cannot may understand it simply as the speaker’s expression of a wish. Naya claims that the use of sentence (41) reflects a metapragmatic strategy of taking various users into consideration. That is, while avoiding being too polite so that the request may not threaten the positive face of the members of the social networking service community, the speaker also indicates negative politeness by indirectly requesting the correction (cf. Brown and Levinson 1987).

Second, Hasegawa (2010: chapter 5) observes that soliloquy sometimes appears in conversations. Observe the following dialogue between a teacher, indicated by H (Higher social status), and a student, indicated by L (Lower social status):

(43) H: Hontoni eigo de-wa kuroushimasu.
  really English Loc-Top am-troubled
  ‘English is sure a pain in the neck!’
  L: Eee, honto desukaa?
  EI true Cop.Pol.Q
  ‘Eh, really?’
  H: Honto, honto.
  true true
  ‘That’s true.’
  L: Hee, sensei demo soonandaa.
  EI teacher also same. Cop.ESFP
  ‘Hmm, even teachers have trouble with it.’
  ( Hasegawa 2010: 158)

Because of their different social statuses, L is required to use honorifics. Nevertheless, in her second turn, *Hee, sensei demo soonandaa*, she uses the plain form. Moreover, the exclamatory interjection *hee* and the exclamatory sentence-final particle *daa* indicate that the utterance is understood as soliloquy ( Hasegawa 2010: 160). As soliloquy is inserted in a conversation, this may be a case where a private expression appears within a public environment at a discourse level. According to Hasegawa, L in (43) strategically uses the soliloquy. While she needs to indicate deference, the use of honorifics necessarily indicates psychological distancing as well. When she wishes to simultaneously express deference and intimacy, she “may temporarily quit the on-going dialogic discourse and switch to soliloquy” ( Hasegawa 2010: 162). Hence, Hasegawa views this strategy as a “metapragmatic shift” by which to mitigate the psychological distancing.

In both cases, the use of a private expression within a public forum may be related to the indication of intimacy or solidarity. Although they are examples in Japanese, the same is true in English. Hirose (2013) observes that sentence (44b) conveys a greater sense of closeness than (44a).

(44) a. I hope you like it, {sir/Professor Brown}.
b. ? Hope you like it, {sir/Professor Brown}.
  ( Hirose 2013: 24)

According to Hirose (2013: 24), by omitting the subject *I*, the speaker of sentence (44b) “is describing the situation not from the perspective of an outside reporter, but from that of an inside participant, which he imposes on the addressee; this results in the speaker bringing the addressee closer to him”. Thus, Hirose points out that the subjectless sentence is not compatible with a respectful form of address, as shown in (44b). This might seem contradictory to the fact that the soliloquy in (43) indicates the speaker’s intimacy to the addressee of a higher social status. However, the soliloquy in (43) serves as a private expression, which is not intended to be communicated to but to be overheard by the hearer. In contrast, because of the address terms, (44b) must be considered a public expression addressed to *sir* or *Professor Brown.* Therefore, they are essentially distinguished from each other. Hirose (2013) observes that (44b) is a subjective utterance compared with (44a), just like sentences typical of diary English, as in (28). Crucially, without the address terms, the subjectless utterance *hope you like it* marks friendliness, and to this extent, the use of soliloquy to be overheard in a conversation and the subjectification by omitting the first-person subject both contribute to shortening the distance between the speaker and hearer.

Generally, by using subjective expressions, the speaker brings the hearer closer to him/her ( Hirose 2013). Private expressions are considered to be highly subjective because they may be used regardless of others (cf. Hirose 2016). In other words, the speaker as a private self reveals his or her “bare” thoughts. The preface to Hasegawa’s (2010) monograph describes the act as follows: “revealing one’s thoughts without interpersonal linguistic devices is a manifestation of trust”. By being attracted to the speaker who reports a situation from the inside, the hearer also has to see the situation from the same perspective as the speaker—namely, the perspective from the inside. Accordingly, a sense of the involvement in the situation may be virtually shared with the hearer, yielding an effect of joint attention. Note that joint attention effects are observed not only in the context of early-stage language acquisition (e.g., Tomasello 1988) but also in certain linguistic uses among adult speakers (e.g., Cheshire 1996). It is also worthwhile pointing out a similarity between some elements in the X-slot (e.g., bare nouns) to what Quine (1969) termed observation sentences, like “Dog!”, where joint attention, or empathy in the Quinean terms, is essential for the understanding thereof. Therefore, indicating closeness or intimacy—and hence inviting the hearer’s empathy—in this manner is a shrewd strategy to get along with others, particularly in the environment of online communication, where nonverbal information such as facial expressions and paralanguage is not available (cf. Naya 2017). ^20^

## 8. Conclusion

This article has claimed that the X-element in the *because* X construction represents the speaker’s private expression while the whole construction functions as a public expression. To account for the two-layered expressive structure, using the notion of small discourse, I have argued that the speaker who starts the discourse with the unmarked mode of expression in English switches his/her perspective to the inside perspective as the discourse progresses. Although these basic claims are in line with Kanetani (2017), the present article has emphasized the significance of the two-layered expressive structure and given an answer to the question left open in the earlier work: what effects are brought about by the proposed two-layered expressive structure. By encapsulating private expression within the public expression, the speaker becomes involved in the situation while avoiding the abruptness. At the same time, by using a subjective expression, the speaker also brings the hearer closer to him/her (cf. Hirose 2013), so that the hearer feels intimacy toward the speaker (cf. Hasegawa 2010; Naya 2017).

I conclude this discussion by comparing the present argument with the treatment of the construction in the previous studies. Bohmann (2016) views the densification of information as a motivation (cf. Biber and Finegan 2001), while Okada (2020) characterizes the X-element as a reference point for the conceptually relevant proposition. These observations seem to account for essentially the same mechanism from different directions. As a reference point, the X-element facilitates the comprehension of the target propositional meaning (cf. Langacker 1993). To do so, the proposition is condensed into the most salient element, which may function as a “keyword” that best represents the proposition. The present conclusion that the X-element is subjective enough to attract the hearer closer to the speaker and accordingly bring about a joint attention effect is compatible with the previous views. As noted in section 7, the X-element is similar to an observation sentence used when the speaker expresses a situation as he/she construes it. When the speaker provides the X-element as a keyword, the hearer empathetically viewing the situation from the situation-internal perspective is ready to recover the intended propositional content based on it. This conclusion also directly supports Bergs’s (2018) observation that the construction is subjective. However, the hearer’s commitment toward what is being said, as well as the speaker’s commitment, is crucially involved.

## Data availability

All data underlying the results are available as part of the article and no additional source data are required.

## Footnotes

^1^
In this article, I do not distinguish spoken language from written (and typed) language and consistently use the term *speaker.* Similarly, I use the terms *hearer*, *addressee*, and *interlocutor* even in contexts of written or computer-mediated communications.

^2^
The survey was conducted from January to February 2014. The results might be different if a survey with the same sentences were conducted now. Since, as Bohmann (2016: 161) notes, the construction is a “rapidly diffusing innovation”, its usage may expand rapidly; accordingly, the acceptability might vary in a different survey.

^3^
Other possibilities of the development of this construction are discussed by Bergs (2018) and Kanetani (2019). Both Bergs (2018) and Okada (2020) reveal that the structure of the *because* X construction, though innovative, is not as new as one might think.

^4^
The term *exclusive subjunct* owes to Quirk *et al.* (1985). For a more complete list of them, see Quirk *et al.* (1985: 604). Detailed semantic differences between exclusives are not considered in this article.

^5^
In Kanetani (2017), assuming that a compressed clause is a register-specific equivalent to a clause, that is, a clause used particularly in computer-mediated communication, I eliminated compressed clauses from the analysis. However, given the considerable frequency of compressed clauses and their differences from full clauses (see section 4.1), I have included them in the present analysis.

^6^

Okada (2020: 8) accounts for this fact in terms of Langacker’s (1993) notion of *reference point*, saying that “elements of any category will appear as far as they work as reference points for the conceptually relevant proposition”.

^7^
It is not clear whether all compressed clauses may be analyzed in this way. More independent research needs to be conducted on the similarities between compressed clauses and interjections.

^8^

Jordan (1989) reports that autistic children use proper names, instead of the first person pronoun, for self-reference. Cappelle (2014) observes an unusual language use by Jerome, a character in the Flemish comic book *Suske en Wiske.* Cappelle relates Jerome’s failure in the proper use of pronouns and his closed eyes as his defensive attitude of cutting off the sight of others’ existence while speaking. These studies also suggest that social interaction with or the assumed presence of others is a prerequisite for the appropriate use of pronouns.

^9^
This seems to rule out *because essay.* However, McCulloch explicitly says that “the *because* + noun construction really must consist of a bare noun, not a noun with a determiner or an adjective”. Therefore, assuming that she simply misplaced the asterisk, I consider *because my essay/an essay/this essay* to be ruled out, but *because essay* to be ruled in.

^10^
Although I do not discuss whether the verb *reset* is used in the infinitival form or the past form, we can tell that the sentence expresses a specific event in the past because of the temporal expressions *for 8* and *for 930*, and the auxiliary verb *could* in the preceding sentence.

^11^

Mendes Junior and Mattos’s (2021: 31) corpus research identifies examples of inflected verbs following *because*, but no examples with a past or third person singular present verb are reported. Mendes Junior and Mattos analyze the verbs in the examples they found either as part of a reduced clause, as a discourse marker (e.g., *see*?), or as a result of the regular deletion of pronoun and auxiliary verb (i.e., the same type as (11a, b)). Many other examples have also been analyzed as verbs or verb phrases following *because* in the literature, including (13), which can be seen as a reduced clause. They should be treated separately in each of the different categories; only those that are neither reduced clauses nor instances of regular deletion, like (23), should be treated as representing the pattern of [ *because* + verb].

^12^

Bergs (2018) suggests that as with nouns, “bare” adjectives seem to be preferred for this construction. By “bare” is meant adjectives with no premodifier like *very* (e.g., *because (?very) unexpected* ( Bergs 2018:48)). However, I leave it for future research as to how this tendency may be related to the privateness of the X-element. I did not consider what may indicate the privateness of the adverbs used in this construction, either. This is also left for future research.

^13^
The abbreviations used in (26) are as follows: PROP1-4 = propositions1-4; POL = polarity; TNS = tense; ASP = aspect; PRED = predicate; ARG _1-n_ = arguments selected by PRED.

^14^
The parentheses are used to indicate where the pronoun *I* is omitted.

^15^
Some reduced clauses used in the *because* X construction omit subjects other than *I*, as exemplified in (i): (i) Those moments when you choose to eat a salad not because you want salad… but because want croutons. (Twitter; cited from Carey 2013, underline added) The omitted subject here is *you.* In Kanetani (2017), this sentence was accounted for in the same way as in the case of diary English. Given the difference in the person of the omitted subject, however, a different explanation should be sought. The absence of an intended addressee (referred to as *you*) in the use of private expressions could account for the omission of *you* in (i) (cf. Hasegawa 2010: chapter 6).

^16^
These are simplified figures. For more detailed figures, see Hirose (2015: 124-125).

^17^

Figures 3 and 4 are reproduced with permission from Taishukan Publishing Co., Ltd.

^18^
The abbreviations used in the glosses of Japanese examples are as follows: 1.Sg. = first person singular pronoun; Acc = accusative marker; Cop = copula; Dat = dative marker; EI = exclamatory Interjection; ESFP = exclamatory sentence final particle; Gen = genitive marker; Imp = imperative form; Loc = locative marker; Nom = nominative marker; Pol = polite form; Q = question particle; SFP = sentence final particle; Super-Pol = super polite form; Top = topic marker.

^19^
The Japanese characters in Figure 4 read as follows: メアリー = *Mearii* ‘Mary’; わたし = *watashi* ‘me’; 話し手 = *hanashite* ‘the speaker’.

^20^
Unlike Naya’s (2017) example of innovative use of *kudasai* ‘please’ in Japanese, the *because* X construction is not restricted to online communications, but as most examples have been documented from online resources, particularly from Twitter ( Bohmann 2016), the environment may well affect or motivate the construction.
