# Supplementary material for: A grammatico-pragmatic analysis of the  because X construction: Private expression within public expression
Source: F1000Res. 2022 Feb 28;10:965. Originally published 2021 Sep 24. [Version 2] doi: 10.12688/f1000research.72971.2 (PMC8902260; doi:10.12688/f1000research.72971.2)
Supplement: Peer review history

# Peer review: Reviewer response for version 2

**DOI:** 10.5256/f1000research.121647.r125627

**Published:** 2022-03-07

**Authors:** Axel Bohmann

**Affiliations:** 1 Albert-Ludwigs-Universität Freiburg, Freiburg, Germany

The author has addressed all my comments in this revised version. While I still feel the treatment of all instances of *because X* as belonging to one construction (even if now differentiated into more or less prototypical cases) may not be the most fruitful approach, the analysis is internally coherent. I have no reservations against this article being accepted in its revised version.

Is the work clearly and accurately presented and does it cite the current literature?

Yes

If applicable, is the statistical analysis and its interpretation appropriate?

Not applicable

Are all the source data underlying the results available to ensure full reproducibility?

No source data required

Is the study design appropriate and is the work technically sound?

Partly

Are the conclusions drawn adequately supported by the results?

Partly

Are sufficient details of methods and analysis provided to allow replication by others?

Partly

Reviewer Expertise:

Sociolinguistics, corpus linguistics, World Englishes

I confirm that I have read this submission and believe that I have an appropriate level of expertise to confirm that it is of an acceptable scientific standard.

# Peer review: Reviewer response for version 1

**DOI:** 10.5256/f1000research.76587.r95402

**Published:** 2021-10-15

**Authors:** Bert Cappelle

**Affiliations:** 1 Laboratoire Savoirs Textes Langage, Université de Lille, Lille, France

**1. General discussion and assessment**

 In this article, Masaru Kanetani presents his original analysis of clauses with *because *X, where X **is usually a single contentful word. Sentence (1) below, from the English Web 2008 corpus (enTenTen08) is a relatively early example:

 (1) Sometimes we have to do all our work over again because flood.

 Given its apparent novelty and idiosyncrasy, this construction has attracted some attention in the recent linguistic literature (cf., e.g., Kanetani 2015 and subsequent publications by the same author, Bohmann 2016, Bergs 2018, Okada 2020, Mendes Junior and Mattos 2021). The construction was, as far as I can see, first noted in the linguistic blogosphere in early July 2012 (Bailey 2012, McCulloch 2012, Liberman 2012) and, as Kanetani mentions, it rose to notoriety when *because *was voted 2013 Word of the Year by the American Dialect Society precisely because of the use at issue here.

 As in some of his previous publications, Kanetani analyzes the construction in terms of Yukio Hirose's three-tier model of language use (cf. Hirose 2013, 2015), which distinguishes between a level of private thoughts, a level of objective reporting, and a level of interpersonal relations. While Hirose's model has been applied here and there to a varied range of grammatical phenomena (cf., e.g., the papers in Ikarashi 2013, part 1; Shizawa and Hirose 2015; Wada 2018, 2019), it is perhaps not yet widely known, nor is it particularly easy to grasp for the non-initiated. Kanetani's paper is commendable in its effort to explain it, not just in words but also with the help of visuals, which I thought was nice.

 For the benefit of the reader of this report, the essence of Hirose's proposal, which Kanetani draws on, is that languages like English and Japanese differ in how the speaker tends to see herself in a speech situation and how she communicates her thoughts to others. Put simply, in English, the speaker ordinarily sees herself objectively, as a 'public self' alongside the other speech participants. This leads to general explicitness in the use of pronouns and accessible NPs, as the speaker is supposed to be aware of the hearer in the outer world and hence should be as clear as possible. At the same time, the speaker does not need to consider her social relationship with the hearer when reporting a situation, which results in few honorifics or other politeness markers. By contrast, in Japanese, the speaker typically looks at the reported situation from within, muttering as it were to herself, which leads to the frequent omission of situationally and contextually retrievable elements (hence fewer pronouns and discourse-familiar NPs); yet, though perceiving herself as a 'private self', the speaker does need to take into account the nature of her relationship with the hearer. This necessitates the encoding of interpersonal aspects, for instance in the form of honorifics, which Japanese speech abounds in. 

 As said, it is against the background of this typological distinction that Kanetani treats the *because *X construction. He claims that, in keeping with the default communicative mode of English, the matrix clause of the construction is public but that the X-element itself is private, thereby giving a glimpse of how the speaker construes a situation for herself, much as in the default mode of Japanese soliloquy-like speech (cf. Hasegawa 2010 on soliloquy phenomena in Japanese and in English). Of course, despite expressing the speaker's thoughts, the X-element *is *ultimately communicated. What is new to Kanetani's present paper, compared to his previous publications on the subject, is that it seeks to provide a (meta)pragmatic, discursive motivation for this public-private shift within a single sentence. While I am not sure whether the term 'metapragmatic' is warranted -- this concept would merit being defined and explained in the text -- Kanetani's proposal makes sense to me. He argues that by slipping into the private, expressive mode, the speaker hopes to connect with the addressee. So, almost paradoxically, by using inward speech (the X-element), the speaker reaches out to the hearer, trying to bond with him.

 The text is well-written, is appropriately structured, and has a well-stocked and up-to-date reference section. The argumentation is coherent and the conclusion is more than sufficiently original. As the paper does not use any new empirical material compared to earlier research by the author or by other authors, the availability of the underlying data is rendered unnecessary. However, as is also noted by Bohmann in his report, this has the downside that the examples used in the text may come across as rather conveniently suiting the analysis put forward. Nevertheless, Kanetani also discusses a few examples that seem to be problematic at first sight (namely examples (38a-b) in the manuscript) and manages to offer an explanation for them. In view of the above unmistakable merits, I recommend the paper be **approved for indexing** and that only minor changes be made to it.

 In what follows, I will spell out some points where I may not fully agree with Kanetani or where I think some clarifications may be in order, without this entailing that the author should revise his analysis in any major way. In at least one case, my points may even lend further support to the crux of his analysis.

**2. Specific points**

 (Page numbers refer to the PDF version of the article).

*2.1  Speech acts in the main clause*

 On p. 4, Kanetani writes about (3c), reproduced below, that "[a] speech-act *because*-clause as in (3c) serves as a motivation for performing *a certain speech act* such as asking about the interlocutor’s plans for the night." (italics added to Kanetani's original)

 (3c) What are you doing tonight, because there’s a good movie on.

 The italicized part seems to suggest that no speech act is performed in the main clauses of (3a) and (3b), while it could be argued that declarative clauses such as *John came back* and *John loved her* also perform a speech act, namely an assertive one. In what sense, then, is the function of the *because*-clause in (3c) any different from its function in (3b), where it provides a reason for making a statement ( *John loved her, because he came back*)? Kanetani does treat Sweetser's (1990) 'speech act' (e.g. (3c)) and 'epistemic' (e.g. (3b)) uses together later on in his article (section 6), so this suggests the difference may not be that important to him.

*2.2  Speech acts in the subclause*

 The discussion of example (6) on p. 6, reproduced below, is to my mind problematic for a couple of reasons.

 (6) *He’s not going out for dinner because Japanese food, his wife is cooking. (Kanetani 2019: 55)

 First, because the example is so utterly bizarre, I found it hard to understand what it is trying to prove. It is meant to illustrate that an unusual speech act is not acceptable in a *because*-clause when this is a cause-expressing adjunct that plays a role in the propositional contents of the whole clause. I am not suggesting that the author should explain more explicitly, as he does in Kanetani (2019), that this example is used to show that you can't insert a topicalization construction in the *because*-clause. After all, I suspect the topicalization construction is not acceptable here on irrelevant, largely information-structural grounds. Moreover, to be convincing, the asterisked example would have to be contrasted with acceptable examples in which the same or a similar *because*-clause has a use in the epistemic/speech-act domain. It would therefore actually be better to use a different kind of performative subordinate clause, in Lakoff's (1987) terms, one that would be more readily interpretable and information-structurally appropriate, for instance:

 (6)' He wasn't going out for dinner because wasn't his wife cooking the best Japanese food he could imagine?

 or

 (6)'' He wasn't going out for dinner because boy could his wife cook fantastic Japanese food!

 Note, by the way, that I have used the past tense, in order to (virtually) rule out the epistemic reading.

 Second, it can be observed that the alternative examples I provide here may not be unacceptable! Consider also this alternative to (3a):

 (3a)' John came back because boy did he love her!

 Here are some authentic, web-attested examples where the *because*-clause is a 'performative subordinate clause' (Lakoff's term again) functioning in the content domain:

 (i) He slept in the crate in the house for that first night because boy did he stink! (www)

 (ii) After Peanut and Marco got back, I cut Walt's hair, and he went in the bathroom and took a bath because boy did he stink. (www)

 (iii) he NEVER takes any advice from kai about dating ever again because boy did he embarrass himself. (www)

 As should be clear, (i) doesn't mean: 'He must have slept..., because...' or 'That I'm making this (perhaps random) statement is justified because...'; in other words, the *because*-clause doesn't have an epistemic/speech-act function. Rather, it mentions the cause of the subject referent (a dog) being confined to sleeping in a crate. Likewise, 'real-world' causal relations hold between the subordinate and main clause situations in (ii) and (iii). Here's another example from the GloWbE corpus (Davies 2013), with full context:

 (iv) While Roarke may not be intimidated by the rancher, Holloway can’t say that he wasn’t a little anxious acting opposite Kevin Costner. “I love working with legends, because boy, does it make you nervous,” the Lost vet says. “I used to hate getting nervous, but somebody told me long ago, you just have to get comfortable being uncomfortable in this business. And I’m like, ‘Oh s–t, I guess you’re right.’ So I don’t get that uncomfortable anymore unless I’m working with a legend.

 “And I like that,” he continues. “It brings your A game, you know? And I’ve been a huge fan of Costner’s forever, so to get on set with him? The first 15 minutes, I’m shakin’ in my boots! Then after you get into it, it’s like, ‘Oh man, this is awesome!’ (GloWbE)

 Here again, the *because*-clause applies to the content domain. Empirical research is likely to confirm, though, that most performative *because*-clauses appear with the epistemic/speech-act function, as in the following example (where the *because*-clause functions epistemically):

 (v) Kay Daly must have eaten a whole bag of lemons because boy, is she sour! (GloWbE)

 In any case, examples (i)-(iv) undermine Kanetani's claim that a content *because*-clause always forms one speech-act unit together with the main clause. In these examples, the speaker simultaneously provides information about why the main clause situation obtained and expresses her emotional involvement about this cause.

 If it is recognized that in the content domain, a *because*-clause can perform its own speech act independently from the speech act of the main clause, then this should mean, as Bohmann also pointed out in his report, that the X in the *because* X construction may also really perform a speech act of its own. 

 As I see it, this should not run counter to the central claim that X is private, quite on the contrary. To the extent that private language is more 'unfiltered' and a more direct representation of the speaker's emotions, we actually *expect* it to have a broader range of speech-act functions than just assertions. The frequent use of interjections and exclamative abbreviations (e.g. *Ugh!*, *Duh!*, *OMG!*, *WTF!*) can then be more readily explained.

*2.3  Playing with perspectives*

 The use of *OMG* (short for *Oh my God*) just mentioned can also combine with a bare noun, as in this example from the web:

 (vi) The 14 '90s Songs You Had Been Most Embarrassed To Take Heed To Together With Your Dad And Mom, Because Omg Sex! (www)

 What is interesting about this example is not just the combination of *Omg* and a bare noun, nor that the *because *X part can have its own speech-act value (note that the exclamation mark belongs to the *because *X **segment, not to the host sequence, which isn't even clausal), but that this segment takes the perspective of the *you *in the title. We get the viewpoint of an imaginary speaker who can be pictured to utter something like, "I just can't listen to this song together with my mom and dad, because Omg sex!"

 In example (iii) above, the perspective in the performative *because*-clause may also be that of the third-person subject in the main clause, not that of the speaker. I think Kanetani should acknowledge this possibility. As it is, he provides an explanation for (38a), which also has a third-person subject but in which the *because *X part is (correctly) argued to represent the speaker's viewpoint. Example (vi) illustrates that the viewpoint can also be someone else's, or perhaps more correctly, the viewpoint can be one that belongs to both someone else and the speaker, which is in line with the idea of solidarity argued for by Kanetani.

*2.4  The boundaries of the construction*

 I came across the following example, from the BNC:

 (vii) but the reason that I got up to ten stone is because christmas 

 Is this an example of the *because *X construction? If this isn't an erroneous utterance, it has the form of a specificational copular pattern "The reason that ... is because X", which is different from the pattern discussed in the text. I do not think it would be possible to use *that *instead of *because*, while this is fully acceptable if *because* introduces a clause:

 (viii) a. but the reason that I got up to ten stone is {because/that} I've been engorging myself on mince pies, turkey stuffing and eggnog over the Christmas holiday

         b. but the reason that I got up to ten stone is {because/*that} christmas

*2.5  Focalized *because X

 The examples given in (9), quoted below, are suspect:

 (9) a. Living people bother you because angry. Ghost make trouble only because sad, lost, confused. (COCA)

       b. If a society needs a large, powerful law enforcement establishment, then there is something gravely wrong with that society; it must be subjecting people to severe pressures if so many refuse to follow the rules, or follow them only because forced. (Corpus of Global Web-Based English [GloWbE])

 Example (9a) is just weird. It's from a novel where a character clearly uses non-native English. Indeed, the whole context is this:

 "Living people always more trouble than ghost," Kwan continued. "Living people bother you because angry. Ghost make trouble only sad, lost, confused." I thought of Elza, pleading for Simon to hear her. "Ghost, I know how catch," said Kwan. "My third auntie teach me how. I call ghost—'Listen me, ghost!'—one heart speaking each other." 

 Example (9b) *is *by a competent speaker of English (namely the Unabomber). Here *only because forced *is similar in structure to *only* { *when*/ *if*} *forced*. It is close to the kind of structure that Kanetani excludes from the analysis ("Therefore, the systematic “subject + copula” deletion structure should be distinguished from Bohmann’s (2016) reduced clause and eliminated from the analysis (at least for the present purposes)" (p.7). This is a reduction of *only because they are forced*. I wonder if there are any real examples of a focalized *because *X.

 By the way, as for the *included* type of reduced clause, it might be interesting to comment on the recursivity apparently allowed by the construction when presenting (13)/(27):

 (13)/(27) Bye going to study for English [ because didn’t finish this morning [because fell asleep]].

 Observe that the underlining should probably include *this morning*.

 In fact, though, I'm no longer sure this is an example of the *because *X construction: note that the main clause, too, is elliptic ( *I'm *is dropped), and so the whole utterance might illustrate private-like language use.

*2.6  Modified *X

 On p. 10, Kanetani writes: "Bergs (2018: 49) [...] reports that “all examples in COCA and COHA have bare nouns” and observes that adding a prenominal modifier or determiner diminishes the acceptability, as shown in (22)."

 This is the example:

 (22) [...] “Because (?favorable/?the) circumstances. I was just lucky, really …”

 This restriction is not obviously explained by the private nature of X, and I can imagine cases where an adjective isn't so bad, especially if it forms a close lexical unit with the noun:

 .

 (ix)  ... because bad breath.

 Here are some further examples from COCA (Davies 2008-)

 (x) ... because young adults.

      ... because heavy rains.

      ... because free speech.

      ... because blonde hair.

      ... because easy access.

      ... because black man.

      ... because Golden Age.

      ... because social programs.

*2.7  Additional/Complementary semantic effects*

 I think the notion of "joint attention" should be explained a bit more fully: how is this established by a private expression and why don't all forms of communication, private or public, establish joint attention on what is being referred to? What does the "hearer's commitment", mentioned in the last sentence of the conclusion, really consist of? Is it a matter of being 'open' to the speaker's privately construed situation? So, how do these semantic (or pragmatic, or even 'metapragmatic' -- to be explained more fully!) effects arise? For the speaker to use language that is *metapragmatic*, I assume she has to use language that explicitly deals with pragmatic effects, which I'm not sure is going on here. The pragmatics of language use have to be discussed via language.

 My impression is that in many of the *because* X cases, the speaker conveys something like 'I know that I'm presenting the reason in a very condensed, almost cryptic way, but I'm sure you know what I mean by it.' Or in simpler terms, 'The reason is X. Need I say more?' There's a sense of 'nuff said' about the X. Especially if X is realized by a noun (with or without modifier), there's a sense of 'You and I know that X inevitably leads to the sort of situation reported in the main clause'. 

*2.8  Suggestions for minor reformulations*

 p. 3: "After outlining the research methodology in section 2, section 3 observes the semantic and syntactic properties of the construction." → dangling participle, so perhaps: "After outlining ... in section 2, I deal, in section 3, with the semantic and syntactic..." or some other sort of solution.

 p. 3: "the description of grammar" → "the description of its grammar"?

 p. 3: "from a perspective of the three-tier model of language use" → "from the perspective..."?

 p. 4: "the real-world" → no hyphen needed, I suppose

 p.5: "The rhetorical question in the *because*-clauses in (5a, b), *isn’t it a beautiful day*, performs a state speech act conveying that it is a beautiful day." → is "state speech act" the term we need, rather than "statement speech act" or something like that?

 p. 5: "merely saying a sentence-final *because*-clause is not sufficient" → "uttering" or "using"?

 p. 5: "this generalization compensates for but is not incompatible with what Lakoff (1987) says" → I'm not sure I understand what is meant here by the underlined sequence; in any case, given the comments above, the paragraph in which this appears may have to be rewritten.

 p. 6: " the focalization is possible for content *because*-clauses but not for epistemic/speech-act *because*-clauses" → drop "the"?

 p. 7: "Reduced clauses also need to be considered." → a little odd that this isn't elaborated further at this point in the text or that there is no reference to where it *is *elaborated.

 p. 10: "English personal pronouns are primarily defined as public expressions that can be diverted to represent the private self" → this by itself is not quite clear enough

 p. 10, footnote 8: "Jerome" → in the original (Flemish) version, the name is rendered as "Jerom".

 p. 11: "a verb of commutating" → communicating, I suppose?

 p. 15/16: "which makes the unmarked mode of expression in English public expression" → I found this hard to process. (Once I saw how it's to be parsed, it was crystal clear, but it wasn't at first)

 p. 16: "Crucially, it is not the whole construction but only its part that deviates from the norm of the English language." → suggested change: "Crucially, it is not the whole construction that is subjective but only the part that deviates from the norm of the English language."

 p. 18: I see both the spelling *tooan *and *touan *(right before and in example (41)) → should there be two different spellings here?

 p. 18: "L in (43) strategically uses the soliloquy" → maybe indicate that this happens in the last turn

 In the references: 

 Carey S: ‘Because’ Has Become a Preposition, because Grammar. A blog post to Science First on November 11, 2013. (retrieved June 21, 2021). → "Sentence First"

 Hirose Y: Deconstruction of the Speaker and the Three-Tier Model of Language Use. Tsukuba English Studies. 2013; 32: 1–28.

Publisher Full Text → the full-text link opens the reference below this one

Is the work clearly and accurately presented and does it cite the current literature?

Yes

If applicable, is the statistical analysis and its interpretation appropriate?

Not applicable

Are all the source data underlying the results available to ensure full reproducibility?

No source data required

Is the study design appropriate and is the work technically sound?

Yes

Are the conclusions drawn adequately supported by the results?

Yes

Are sufficient details of methods and analysis provided to allow replication by others?

Yes

Reviewer Expertise:

English grammar, cognitive linguistics, Construction Grammar, corpus linguistics

I confirm that I have read this submission and believe that I have an appropriate level of expertise to confirm that it is of an acceptable scientific standard.

## References

[1] : Because reasons. a blog post to linguistlaura (July 2, 2012).2012 (retrieved October 13, 2021); Reference source

[2] : Because Science!: Notes on a Variable Conjunction In: Seoane E, Acuña-Fariña C, Palacios-Martínez I (eds.) Subordination in English: Synchronic and Diachronic Perspectives. *Berlin: Walter de Gruyteryear* . 10.1515/9783110583571-003 43-60 10.1515/9783110583571-003

[3] : Language Change because Twitter?: Factors Motivating Innovative Uses of because across the English-Speaking Twittersphere. In: Lauren S (ed.) English in Computer-Mediated Communication: Variation, Representation, and Change. *Berlin: Walter de Gruyter* .2016; 10.1515/9783110490817-008 149-178 10.1515/9783110490817-008

[4] : Peer Review Report For: A grammatico-pragmatic analysis of the because X construction: Private expression within public expression [version 1; peer review: 1 approved with reservations]. *F1000Research* .2021;10(965) : 10.5256/f1000research.76587.r95428 10.5256/f1000research.76587.r95428

[5] : The Corpus of Contemporary American English (COCA).2008; Reference source

[6] : Corpus of Global Web-Based English (GloWbE).2013; Reference source

[7] : Soliloquy in Japanese and English. *Amsterdam: John Benjamins* .2010; 10.1075/pbns.202 10.1075/pbns.202

[8] : Deconstruction of the Speaker and the Three-Tier Model of Language Use. *Tsukuba English Studies* .2013;32:1-28

[9] : An Overview of the Three-tier Model of Language Use. *English Linguistics* .2015;32(1) : 10.9793/elsj.32.1_120 120-138 10.9793/elsj.32.1_120

[10] : Part I : Papers on the Three-Tier Model of Language Use. *Tsukuba English Studies* .2013;32:1-144 Reference source

[11] : On the New Usage of Because. *Studies in Language and Literature [Language]* .2015;68:63-80 Reference source

[12] : Causation and Reasoning Constructions. *Amsterdam: John Benjamins* .2019; 10.1075/cal.25 10.1075/cal.25

[13] : Women, Fire and Dangerous Things: What Categories Reveal about Mind. *Chicago: University of Chicago Press* .1987;

[14] : Because NOUN. a blog post to Language Log (July 12, 2012).2012 (retrieved October 13, 2021); Reference source

[15] : Because Reasons. a blog post to All Things Linguistic on (July 4, 2012).2012 (retrieved October 13, 2021); Reference source

[16] : “[Because X] sob a perspectiva da Gramática de Construções: uma análise baseada em corpus” (Understanding [because X] in Construction Grammar: A Corpus-Based Analysis). *Domínios de Lingu@gem* .2021;15(4) :1-42 Reference source

[17] : Category-free complement selection in causal adjunct phrases. *English Language and Linguistics* .2020; 10.1017/S1360674320000295 1-23 10.1017/S1360674320000295

[18] : Introduction: public/private-self-centeredness and grammatical phenomena in Japanese and English--The perspective of the Three-Tier Model of Language Use. *English Linguistics* .2015;32(1) : 10.9793/elsj.32.1_114 114-119 10.9793/elsj.32.1_114

[19] : From Etymology to Pragmatics: Metaphorical and Cultural Aspects of Semantic Change. *Cambridge: Cambridge University Press* .1990; 10.1017/CBO9780511620904 10.1017/CBO9780511620904

[20] : C-gravitation and the grammaticalization degree of “present progressives” in English, French, and Dutch. In: Hancil S, Breban T, Lozano, JV (eds.), New Trends in Grammaticalization and Language Change. *Amsterdam: John Benjamins* .2018; 10.1075/slcs.202.09wad 207-230 10.1075/slcs.202.09wad

[21] : The Grammar of Future Expressions in English. *Tokyo: Kaitakusha* .2019;

# Response

**Published:** 2022-02-23

**Authors:** Masaru Kanetani

**Affiliations:** University of Tsukuba, Japan

Dear Dr. Bert Cappelle,

 First and foremost, thank you very much for kindly accepting the review request.

 I appreciate your positive assessment of my article and your detailed comments. I also thank you for your brief introduction to the three-tier model of language use, which will be of help for those who are not familiar with the model.

 As there are many things to take into consideration, I have taken a long time to revise the manuscript. I apologize that I couldn't respond to your comments earlier. I have finished revising the manuscript, which will appear shortly after editorial processes. Your comments are helpful in developing the argument in the revised version. Thank you once again for your comments on an earlier version.

# Peer review: Reviewer response for version 1

**DOI:** 10.5256/f1000research.76587.r95428

**Published:** 2021-10-08

**Authors:** Axel Bohmann

**Affiliations:** 1 Albert-Ludwigs-Universität Freiburg, Freiburg, Germany

This article proposes a novel grammatical-pragmatic analysis of a recent innovation in English, viz. the use of *because* followed by a complement other than a prepositional phrase headed by *of* or a finite clause. This usage has come to be referred to as “ *because* X” in the literature. Citing examples from the author’s own previous work as well as other sources, and occasionally additional corpus data, the author proposes an analysis along the following lines:

  

- *because* X only occurs with real-world causality meanings (as opposed to epistemic causality or speech-act justification, following Sweetser’s (1990: 77) ternary distinction).
- The element in the X-slot serves as a “private expression” (following Hirose’s (2000) distinction between “public” and “private expressions”) and hence is atypical for a language like English, in which “the unmarked mode of expression is public expression” (p. 14, page numbers refer to the PDF version of the article)
- This private expression is embedded within the larger structure of a public expression (the superordinate clause of *because* X) in order to allow English speakers to switch to a more private mode of expression in the same “small discourse” and thus not to violate the default expectation of public expression.
- The pragmatic motivation for this two-layered structure is to create a sense of immediacy and involvement while avoiding “abruptness” that allegedly would result from starting an utterance in the mode of private expression.

The article is well-written and clearly structured. The sections themselves are internally coherent, and each section articulates a clear argument. One suggestion I have in terms of structure is to establish the theoretical background earlier and with more detail. Being unfamiliar with Hirose’s models, I did not always feel like I could understand the criteria by which expressions are categorized into “private” and “public”, nor whether these two levels exist to some extent in all utterances or are strictly categorical, mutual opposites. Given that these categorizations are central to the arguments in the paper, but that the theory behind them is, to my knowledge, not widely known, it would really strengthen the paper if the author could provide more clarity here.

 The author is clearly an expert on the *because X *construction, which shows in the careful consideration of all the existing literature on the topic. A comprehensive range of research in three different languages is not only cited, but actively addressed throughout the paper. The author is evidently very careful in achieving a synthesis of all that has been said on the construction so far and is doing a very thorough job in this regard. At times, my feeling is that the author treats the evidence in the literature a bit selectively to suit his line of argument. For example, on p. 10 the paper makes a case that a noun in the X-slot “should be a bare noun” and cites McCulloch (2012) in support of this. Bergs (2018) is also cited, who does present premodification of elements in the X-slot as potentially questionable, but is more careful about this claim than the paper under review gives the impression. Finally, whereas both Schnoebelen (2014) and Bohmann (2016) are discussed in detail elsewhere, both of these contain examples of premodified nouns in the X-slot that the paper is silent about. I would invite the author to address such evidence more actively rather than ignore it.

 A similar point relates to method. Coming from a quantitative-empirical background, I sometimes have a difficult time accepting general conclusions about what is or is not permissible based on isolated examples, some of which are made up rather than being attested usage. Especially with a rapidly diffusing innovation, there is reason to be skeptical of native (or non-native) speaker intuitions as reliable sources of evidence. Isolated corpus examples can offer illustrative evidence, but to make strong claims about what can and cannot occur in the X-slot, more systematic evidence would be desirable. Here are a couple of claims from the paper where I think some of the limited data I collected on the topic in the past provide problematic, if not counter-evidence. (Examples are drawn from the corpus data for Bohmann (2016). I would be happy to make the data available upon request):

  

- p. 5: “As an epistemic/speech act *because*-clause performs a speech act of its own, the *because* X construction is naturally incompatible with an epistemic/speech-act reason clause, because the word or phrase that appears in the X-slot cannot perform an independent speech act.”
  → “ I need to just show you the pic cause hahahahaa” (Twitter, Australia)
  → “I'm still gonna read all of it anyways though because why not” (Twitter, Canada)
  → “Watching old @user videos because why not.” (Twitter, USA)
  → “taeyeon is so lucky because goddammit” (Twitter, Singapore)
  → “Kinda don't wanna do this morning shift because you know” (Twitter, USA)
- The claim, mentioned above, that a noun in the X-slot should be bare:
  → “I'd turn my light off purely because the bad acting” (Twitter, Australia)
  → “In that case i need sex then cos this fever” (Twitter, UK)
- “no examples with a past or third person singular present verb are reported” (fn 11, p. 11)
  → “idk if i am bc depends on baby” (Twitter, NZ)
  → “@user @user oil cos wanted it for the carve up” (Twitter, UK)
  → “Having to squeeze my giant feet into my moms tiny shoes because forgot shoes” (Twitter, USA)
  → “Can't sleep cause stomach hurts” (Twitter, USA)

I do not believe the counterexamples necessarily invalidate the general argument the paper is making, but they indicate that perhaps the claims need to be hedged a bit. If the author is willing to give up treatment of *because X* as one unified construction and instead differentiate between more or less protoypical uses, then I think the argument still holds up (although once again, a more thorough presentation of Hirose’s model would help).

 Despite these reservations at the methodological level, I find a lot of merit in the analysis. On p. 19, the author writes on the insertion of private expressions: “By being attracted to the speaker who reports a situation from the inside, the hearer also has to see the situation from the same perspective as the speaker — namely, the perspective from the inside. Accordingly, a sense of the involvement in the situation may be virtually shared with the hearer, yielding an effect of joint attention.” This, in my view, is a very elegant summary of some of the prototypical effects *because* X is enlisted for. Arguments in a similar direction have been made about quotative BE *like*, which the author may find helpful (e.g. Coupland 2007: 186-187).

 I recommend this article be indexed with moderate revisions, chiefly:

  

- Establish the theoretical background, particularly Hirose’s three-tiered model of language use, and the role of public and private expression in it, earlier and in sufficient detail for the uninitiated reader to follow the analysis.
- Consider the disconfirming evidence (examples given above) for some of the central claims more actively and reframe the analysis accordingly.

Individual comments:

  

- Section 3.2 is introduced as discussing “functional properties” but is entitled “semantic properties”: is semantic and functional the same in this context?
- p. 4: the presentation of examples from Sweetser is only a minimal paraphrase of the original. Consider rewriting to avoid allegations of plagiarism.
- p. 5: Example (6) very hard to make sense of; I find it anything but clear that the matrix negation scopes over the whole sentence, simply because the sentence on the whole is off.
- p. 5: “In short, the use of [ *because* *of* NP] is restricted to the content domain for essentially the same reason as the [ *because* X] being limited to the content reading. That is, neither the NP that follows *because* *of* nor the word that directly follows *because* can perform an independent speech act.” I don’t see enough evidence for this categorical claim (and indeed think counter-evidence is not difficult to find; see above).
- p. 5: Example (9)a) is from a piece of literary writing where the voice of the speaking character is stylized to sound ungrammatical (look at the wider context in COHA). As such, this is a poor choice to say anything about what is or isn’t permissible in regular use.
- p. 6: “The ungrammaticality of (10) shows that the exclusive *just* cannot focalize an epistemic because-clause.” I am not sure if the (claimed) ungrammaticality of an individual, constructed example is sufficient evidence to claim something is generally not possible in the language. See also the attested example given above with “purely because the bad acting”.
- p. 6: “Up to this point, it has been shown that the *because* X construction syntactically behaves like the content *because*-clause construction and not like the epistemic/speech-act *because*-clause constructions.” I think there is a confusion of levels of analysis here. *Because*-clause is accorded multiple functions for the same formal construction, whereas this level of variation does not seem to be granted to *because* X. The argument then is that a) *because* X (also) occurs in contexts which are only permissible in the content reading, and b) therefore ALL of *because* X entails context reading. If one is willing to consider the possibility that there are multiple *because* Xs, then the argument becomes problematic.
- p. 7: “In short, the pronoun is a marginal (if not impossible) category as an X-element.” In the preceding paragraph, the paper references the 2.45% pronouns attested in the X-slot in Schnoebelen (2014) ^5^. So while they play a minor role, hinting at categorical impermissibility is uncalled for, I think.
- p.8: should “section 3” be “section 3.3”?
- p. 10: “Recall that pronouns are not used in this construction.” So now we have come from 2.45% attested pronouns in Schnoebelen (2014) ^5^ to “marginal (if not impossible)” to “are not used.” This selective interpretation of the available evidence is problematic.
- p. 10: “In short, only relatively to others can the personal pronoun be defined and used” (→ “only relative” or “only in relation to”?)
- p. 10: Claimed impermissibility of complex NPs: see counter-examples above, as well as in the literature the paper cites.
- p. 10: Example (24) is not really a felicitous rendition of the meaning of (23). The causality is not “because I slept again” but “because I needed/wanted to sleep” or something similar.
- p. 14: “English speakers prefer to take an objective perspective from outside of the situation while Japanese speakers prefer to take a subjective perspective from the inside.” Those are extremely sweeping cultural claims; more evidence (and more background on the model) would be very useful here.
- p. 16: In section 6, what is “a discourse”, “a scene”? These terms seem to be sufficiently underdefined to accommodate various kinds of interpretation.
- p. 19: “Generally, by using subjective expressions, the speaker brings the hearer closer to him/her” (→ “himself/herself”).

Is the work clearly and accurately presented and does it cite the current literature?

Yes

If applicable, is the statistical analysis and its interpretation appropriate?

Not applicable

Are all the source data underlying the results available to ensure full reproducibility?

No source data required

Is the study design appropriate and is the work technically sound?

Partly

Are the conclusions drawn adequately supported by the results?

Partly

Are sufficient details of methods and analysis provided to allow replication by others?

Partly

Reviewer Expertise:

Sociolinguistics, corpus linguistics, World Englishes

I confirm that I have read this submission and believe that I have an appropriate level of expertise to confirm that it is of an acceptable scientific standard, however I have significant reservations, as outlined above.

## References

[1] : Because Science! Notes on a Variable Conjunction. In: Seoane E, Acuña-Fariña C, Palacios-Martínez I (eds.) Subordination in English: Synchronic and Diachronic Perspectives. *Berlin: Walter de Gruyter* .2018; https://doi.org/10.1515/9783110583571-003 43-60 10.1515/9783110583571-003

[2] : Language change because Twitter? Factors motivating innovative uses of because across the English-speaking Twittersphere.2016; 10.1515/9783110490817-008 10.1515/9783110490817-008

[3] : Style: Language Variation and Identity (Key Topics in Sociolinguistics). *Cambridge: Cambridge University Press* .2007;

[4] : Nihongo kara mita nihonjin: Shutaisei-no gengogaku (Japanese People as Seen from the Japanese Language: The Linguistics of Subjectivity). *Tokyo: Kaitakusha* .2010;

[5] : Because Reasons. a blog post to All Things Linguistic on (July 4, 2012).2012 (retrieved June 21, 2021); Reference source

[6] : Innovating Because Innovation. a blog post to Corpus Linguistics on January 15, 2014.2014; Reference source

[7] : From Etymology to Pragmatics.1990; 10.1017/CBO9780511620904 10.1017/CBO9780511620904

# Response

**Published:** 2022-02-23

**Authors:** Masaru Kanetani

**Affiliations:** University of Tsukuba, Japan

Dear Dr. Axel Bohmann,

 First and foremost, thank you very much for kindly accepting the review request.

 I appreciate your positive assessment of my article and your detailed comments. I am also grateful for providing me with new data.

 As there are many things to take into consideration, I have taken a long time to revise the manuscript. I apologize that I couldn't respond your comments earlier. I have finished revising the manuscript, which will appear shortly after editorial processes. Your comments are helpful in developing the argument in the revised version. While reacting to your comments in the article, I'd like to express my gratitude to you once again.
